# Analysis of glucose metabolism by ^18^F-FDG-PET imaging and glucose transporter expression in a mouse model of intracerebral hemorrhage

**DOI:** 10.1038/s41598-021-90216-4

**Published:** 2021-05-25

**Authors:** Xiaoning Han, Honglei Ren, Ayon Nandi, Xuanjia Fan, Raymond C. Koehler

**Affiliations:** 1 Department of Anesthesiology and Critical Care Medicine, School of Medicine, Johns Hopkins University, Baltimore, MD 21205 USA; 2 Division of Nuclear Medicine and Molecular Imaging, The Russell H. Morgan Department of Radiology and Radiological Science, School of Medicine, Johns Hopkins University, Baltimore, MD 21205 USA

**Keywords:** Neurological disorders, Neuroscience

## Abstract

The relationship between cerebral glucose metabolism and glucose transporter expression after intracerebral hemorrhage (ICH) is unclear. Few studies have used positron emission tomography (PET) to explore cerebral glucose metabolism after ICH in rodents. In this study, we produced ICH in mice with an intrastriatal injection of collagenase to investigate whether glucose metabolic changes in ^18^F-fluoro-2-deoxy-D-glucose (FDG)-PET images are associated with expression of glucose transporters (GLUTs) over time. On days 1 and 3 after ICH, the ipsilateral striatum exhibited significant hypometabolism. However, by days 7 and 14, glucose metabolism was significantly higher in the ipsilateral striatum than in the contralateral striatum. The contralateral hemisphere did not show hypermetabolism at any time after ICH. Qualitative immunofluorescence and Western blotting indicated that the expression of GLUT1 in ipsilateral striatum decreased on days 1 and 3 after ICH and gradually returned to baseline by day 21. The ^18^F-FDG uptake after ICH was associated with expression of GLUT1 but not GLUT3 or GLUT5. Our data suggest that ipsilateral cerebral glucose metabolism decreases in the early stage after ICH and increases progressively in the late stage. Changes in ^18^F-FDG uptake on PET imaging are associated with the expression of GLUT1 in the ipsilateral striatum.

## Introduction

Approximately 10–15% of all strokes are caused by intracerebral hemorrhage (ICH). With few treatment options, however, ICH is associated with high rates of morbidity and mortality^1^. ICH results from the rupture of cerebral blood vessels, which leads to a rapid increase in intracranial pressure and hematoma expansion, followed by secondary brain injury such as edema, cytotoxicity, and inflammation. Treatment strategies being tested in animal models of ICH include promotion of hematoma clearance, inhibition of edema expansion, reduction of secondary inflammation, and reversal of cell death^2^. Recently, increasing research has focused on brain metabolism after ICH, including nitrogen metabolism, iron homeostasis, and energy supply, which are thought to mediate neuronal dysfunction^3–5^.

Glucose is the predominant fuel used by the brain to manufacture ATP. It is delivered to the brain from blood vessels primarily via glucose transporter (GLUT) 1, which is present on the endothelium and astrocytic endfeet^6^.Dynamic stabilization of glucose utilization in brain is critical for neuronal physiologic activity. Blood vessel rupture during ICH disturbs normal glucose delivery and metabolism and has been linked to neurologic deficit as well as cell death and inflammation in the brain^7^. Critical care guidelines state that maintenance of normoglycemia is imperative for ICH patients^8^. However, cerebral glucose is not as frequently measured after ICH as blood glucose is. Using intracerebral microdialysis, several clinical studies have demonstrated that brain glucose concentration gradually declines, whereas concentrations of brain pyruvate and lactate increase during the 7 days after subarachnoid hemorrhage^9,10^. These changes are correlated with brain edema and worse outcome^11^. Conversely, patients with deep ICH failed to show significant changes in glucose, lactate, and pyruvate^12^.

In contrast to intracerebral microdialysis, ^18^F-fluoro-2-deoxy-D-glucose (FDG)-positron emission tomography (PET) is a noninvasive method used to evaluate brain glucose metabolism in patients. ^18^F-FDG is a glucose analog radiopharmaceutical that cannot undergo glycolysis when taken up by live cells^13^. Thus, it can be used to measure glucose utilization and cell viability not only in primary lesions but also in distal brain regions not directly damaged. Previous clinical data have shown that patients with traumatic brain injury had significantly less ^18^F-FDG uptake in the cortex than did normal controls. Moreover, the deficits of glucose metabolism can spread over multiple brain areas, such as into white matter and thalamus, though these regions appear normal in TBI^14,15^.

Although PET scans are considered the “gold standard” for evaluating early stroke pathophysiology^16^, they are not universally applied because of radiation, high cost, and limited availability^17^. Accordingly, little is known about dynamic glucose metabolism after ICH. In a previous study Albers et al.^18^ reported that patients exhibited transiently increased ^18^F-FDG uptake at 2 to 4 days after acute ICH. Similarly, in an autologous blood-induced cat model of ICH, ^18^F-FDG intensity steadily increased from a nadir at 12 h and returned to control level 5 days after ICH^19^. However, patients with subcortical aphasia after ICH showed diffuse hypometabolism and hypermetabolism in frontal, parietal, and temporal cortices^20^. Another recent study showed that patients with chronic neuropathic pain after thalamic ICH also exhibit hypometabolism and hypermetabolism in various brain regions related to sensory processing and cognitive functioning ^21^. Thus, ^18^F-FDG-PET studies of clinical and animal ICH are heterogeneous regarding the localization and time points assessed, and the temporospatial changes in glucose metabolism with PET imaging remain unclear.

In this study, we investigated cerebral glucose metabolism with ^18^F-FDG-PET scans over multiple time points in a collagenase-induced mouse ICH model. Our goal was to ascertain whether the changes in glucose metabolism are associated with the expression of GLUTs after ICH. This study may provide preclinical data for glucose management of patients with ICH.

## Material and methods

### Animals and study design

All experimental procedures were approved by the Institutional Animal Care and Use Committee at Johns Hopkins University School of Medicine and followed the ARRIVE (http://www.nc3rs.org.uk/arrive), STAIR, and RIGOR guidelines^18,22^. Eight- to 10-week-old (23–25 g) C57BL/6 male mice were obtained from Charles River Laboratories (Frederick, MD). A total of 108 mice were used. A power analysis based on our pilot PET data indicated that nine mice/group would provide at least 80% power for detecting a 20% decrease in ^18^F-FDG uptake at α = 0.05 (two-sided test). Animals were randomly assigned for testing at different time points by using the randomizer form at randomizer website. Different investigators were blinded to surgery, data collection, and analysis. Assuming a normal data distribution, we used statistical software (GraphPad Grubbs Test) to define outlying data points and excluded them from the data set. Animals that died during surgery or shortly after ICH were excluded from analysis. Animals that had a neurologic deficit score greater than 20 at day 1 after surgery were euthanized under deep anesthesia.

### ICH model

We used PET scans to assess ^18^F-FDG uptake in mice after collagenase-induced ICH. Anesthesia was induced with inhaled 3% isoflurane and maintained with 1% isoflurane during surgery. The left striatum of each mouse was injected with 0.5 µL of 0.075 U collagenase VII-S (Sigma-Aldrich) at a rate of 0.1 µL/min. The needle was kept in place for 5 min and then removed slowly. The stereotactic coordinates of injection were 0.8 mm anterior and 2.0 mm lateral of the bregma, and 3.0 mm in depth^23^. Sham-operated mice underwent the same protocol but without collagenase injection. Rectal temperature was maintained at 37.0 ± 0.5ºC throughout the experimental and early recovery periods (DC Temperature Controller 40–90-8D; FHC Inc., Bowdoin, ME). Mice underwent PET scans on days 1, 3, 7, 14, and 21 after collagenase injection.

For the platelet immunohistochemistry, we also injected in the left stratum with 10 µl autologous blood at 0.5 µL/min. Mice were perfused for immunostaining on day 1 after collagenase or blood-induced ICH.

### PET scans

All PET scans were performed with the SuperArgus PET/computerized tomography (CT) small animal scanner (Sedecal, Madrid, Spain), part of the Small Animal Imaging Resource Program at the Johns Hopkins Hospital PET Center.

The mice were fasted overnight, anesthetized with 2% inhaled isoflurane gas, and then maintained under 1% isoflurane gas during the entire procedure. An intravenous catheter was inserted into the tail vein, through which 200–250 µCi of ^18^F-FDG was injected. Scanning began immediately after tracer injection, with a 60-min dynamic acquisition. The mice were placed side-by-side, two per scan. Because of the scanner bed design and the anesthesia tube, mice were placed in “best-fit” positions—as close to upright as possible with a slight tilt of the head so that the nose could be placed snugly into the anesthesia tube. This best-fit position ensured that the mice were under anesthesia during the entire 60-min scan. We acquired high-resolution research CT images to allow for localization and anatomic reference. After imaging, mice were kept under a heat lamp until they were fully awake and then placed in an isolation room for 24 h to eliminate radiation hazards.

PET/CT image reconstruction: After acquisition, PET images were histogrammed and reconstructed into 30 dynamic frames. An iterative PET reconstruction was done using the 3D ordered-subset expectation maximization algorithm (3D-OSEM). Images were corrected for attenuation (CT-based), and corrections for scatter and dead-time were applied^24^.

PET images were analyzed with a previously developed data analysis package for mouse brain PET studies^25^. The package uses a prepared template of standard volumes of interest (VOIs). The template was created from high-resolution MRI scans of sham mice (Bruker 9 T MRI). VOI regions in left and right hemisphere include striatum, hippocampus, thalamus, and cortex, and the whole cerebellum and brainstem. The template VOIs were spatially aligned to an individual mouse’s upright CT (reoriented to have a perpendicular mid-plane) according to the mouse’s CT-to-standard CT spatial normalization parameters (i.e., skull-to-skull). We obtained normalization parameters using the software package Statistical Parametric Mapping (SPM, London) and its normalization module. Separately, PET frames were spatially aligned to the upright CT according to PET-to-CT coregistration parameters obtained with an SPM5 coregistration module that employs the mutual-information theory^26^. After confirming that these prepared VOIs agreed with the radioactivity distribution of averaged PET, we applied the VOIs to PET frames to obtain tissue time activity curves (TACs). These TAC curves were converted to standardized uptake value (SUV) units, and the SUV ratio (SUVR) was calculated for each region and time point by using cerebellum as the reference region. The mean SUVR from 40 to 60 min was the main outcome variable. ^18^F-FDG SUVR values of the left (ipsilateral) hemisphere were compared to those of the right (contralateral) hemisphere in treated and sham groups for different brain regions. We normalized averaged PET images to cerebellum values to visualize the changes in FDG-PET uptake in individual mice.

### Immunofluorescence

At each time point after ICH induction, mice were perfused with phosphate-buffered saline followed by 4% paraformaldehyde. For brain hematoma images, brains were cut into 1-mm-thick coronal sections with a mouse brain slicer matrix (Zivic Instruments, Pittsburgh, PA). For immunohistochemistry, brains were post-fixed in 4% paraformaldehyde overnight and then transferred to 30% sucrose. Brains were cut into 30-µm-thick coronal sections for immunofluorescence staining as previously described^27^. The primary antibodies were anti-GLUT1 (1:100, Millipore, 07–1401), anti-CD31 (1:200, Abcam, ab32457), anti-Microtubule-associated protein 2 (MAP2, 1:5000, Abcam, ab5392), anti-NeuN (1:100, Millipore, MAB377), anti-GLUT5 (1:100, Life Technologies, MA1-036 and 1:100, Santa Cruz Biotechnology, sc271055), anti-Glial fibrillary acidic protein (GFAP, 1:500, Sigma, G3893), anti-CD41 (1:1000, Abcam, ab33661), anti-Iba1 (1:500, Wako, 019–19,741), and anti-myeloperoxidase (MPO, 1:200, Dako, A039829-2). The sections were incubated with secondary antibodies (Alexa Fluor 488, Alexa Fluor 594 and Alexa Fluor 680, 1:1000; Molecular Probes, Eugene, OR) for 1 h at room temperature. Nuclei were labeled with DAPI (1:1000, Life Technologies, R37606). Omission of the primary antibody was used as a control. Images were observed under a Nikon Eclipse 90i fluorescence microscope and analyzed with ImageJ software (NIH, 1.47t). Images were captured from three optical fields (20 × 10 magnification) in each of three sections per animal. The average fluorescence intensity was expressed as a percentage normalized to the corresponding section of the sham group^23^.

### Western blot analysis

At different time points after ICH, mice were exsanguinated by perfusion with phosphate-buffered saline. Ipsilateral and contralateral striatum were isolated and homogenized by T-PER reagent (Pierce) with protease inhibitor cocktail (Roche Molecular Biochemicals). Proteins were separated by sodium dodecyl sulfate–polyacrylamide gel electrophoresis and transferred onto polyvinylidene fluoride membranes^28^. Membranes were probed with primary antibodies against GLUT1 (1:1000, Millipore, 07–1401), GLUT3 (1:1000, Santa Cruz Biotechnology, sc-74399), GLUT5 (1:1000, Life Technologies, MA1-036), ZO-1 (1:1000, Life Technologies, 617,300), claudin-5 (1:1000, Life Technologies, 352,500 and 1:1000, Abcam, ab131259), and β-actin (1:3000, Santa Cruz Biotechnology, sc-47778) at 4ºC overnight. The membranes were incubated with horseradish peroxidase-linked anti-rabbit or anti-mouse secondary antibody (1:3000, Santa Cruz Biotechnology) for 1 h at room temperature. Protein signals were visualized in chemiluminescence solution and exposed under an ImageQuant ECL Imager (GE Healthcare). Images were analyzed by ImageJ. Optical density values were normalized to the corresponding loading control intensity and expressed as fold change ^23,29^.

### Flow cytometry

On day 1 after ICH, mice were anesthetized with 3% isoflurane and were perfused with cold phosphate-buffered saline. Brains were cut into 1-mm-thick coronal sections with a mouse brain slicer matrix (Zivic Instruments, Pittsburgh, PA). Four 1-mm-thick brain sections of the left striatum with hemotoma were collected. The striatum was dissociated with GentleMACS Dissociator (Miltenyi Biotec, Auburn, CA) as described previously^28,30^. The final cell suspension were incubated with the primary antibodies: CD11b-FITC (Miltenyi Biotec, 130–113-234) and ACSA-2-APC (Miltenyi Biotec, 130–117-535) or and Ly6G-APC (Pharmingen, 560,599) for 30 min at 4ºC. The corresponding isotype antibodies were used as negative control. Propidium iodide (Sigma, St. Louis, MO) staining was used to exclude dead cells. Cell supernatants were analyzed by CytoFLEX cytometer (Beckman Coulter, Indianapolis, IN) with CytExpert software 2.0 (Beckman Coulter). Macrophage/microglia cells were distinguished as CD11b-FITC^+^ / ACSA-2-APC^-^ population. Astrocytes were distinguished as CD11b-FITC^-^ / ACSA-2-APC^+^ population. Neutrophils were distinguished as CD11b-FITC^+^ / Ly6G-APC^+^ population. Gated cells were collected into TRIzol reagent (Qiagen, 217,004) for mRNA extraction and real-time PCR.

### Real-time PCR

mRNA extraction was performed with the miRNeasy Mini Kit instruction (Qiagen, 217,004). 250 ng of mRNA from each sample was transcribed into cDNA with the SuperScript VILO cDNA Synthesis Kit (ThermoFisher Scientific, 11,754). The following TaqMan gene probes were used to quantify gene expression: GLUT1 (Mm00600697_m1), GLUT3 (Mm00441483_m1) and GAPDH (Mm99999915_g1). The GAPDH was used as internal control. Real-time PCR was performed on QuantStudio3 System (Thermo Fisher Scientific, Waltham, MA) with a hold time of 10 min at 95ºC, and cycled at 95ºC for 15 s and 60ºC for 1 min, for 40 cycles totally. The fold change of GLUT1 and GLUT3 expression in neutrophils were normalized to the sham macrophage/microglia group. The fold change of GLUT1 in astrocytes was normalized to the sham astrocytes group.

### Statistical analysis

All analyses were carried out with SigmaPlot 12.5 software. A probability value of *p* < 0.05 was considered statistically significant. Differences among multiple groups were analyzed by one-way or two-way ANOVA with Bonferroni post hoc test. Pearson’s correlation test was used to evaluate the association between SUVR and GLUT expression^31^.

### Ethical approval

All applicable international, national and institutional guidelines for the care and use of animals were followed. This article does not contain any studies with human participants performed by any of the authors.

## Results

### ^18^F-FDG glucose uptake in the ipsilateral striatum decreases in early stage of ICH

**Figure 1:**
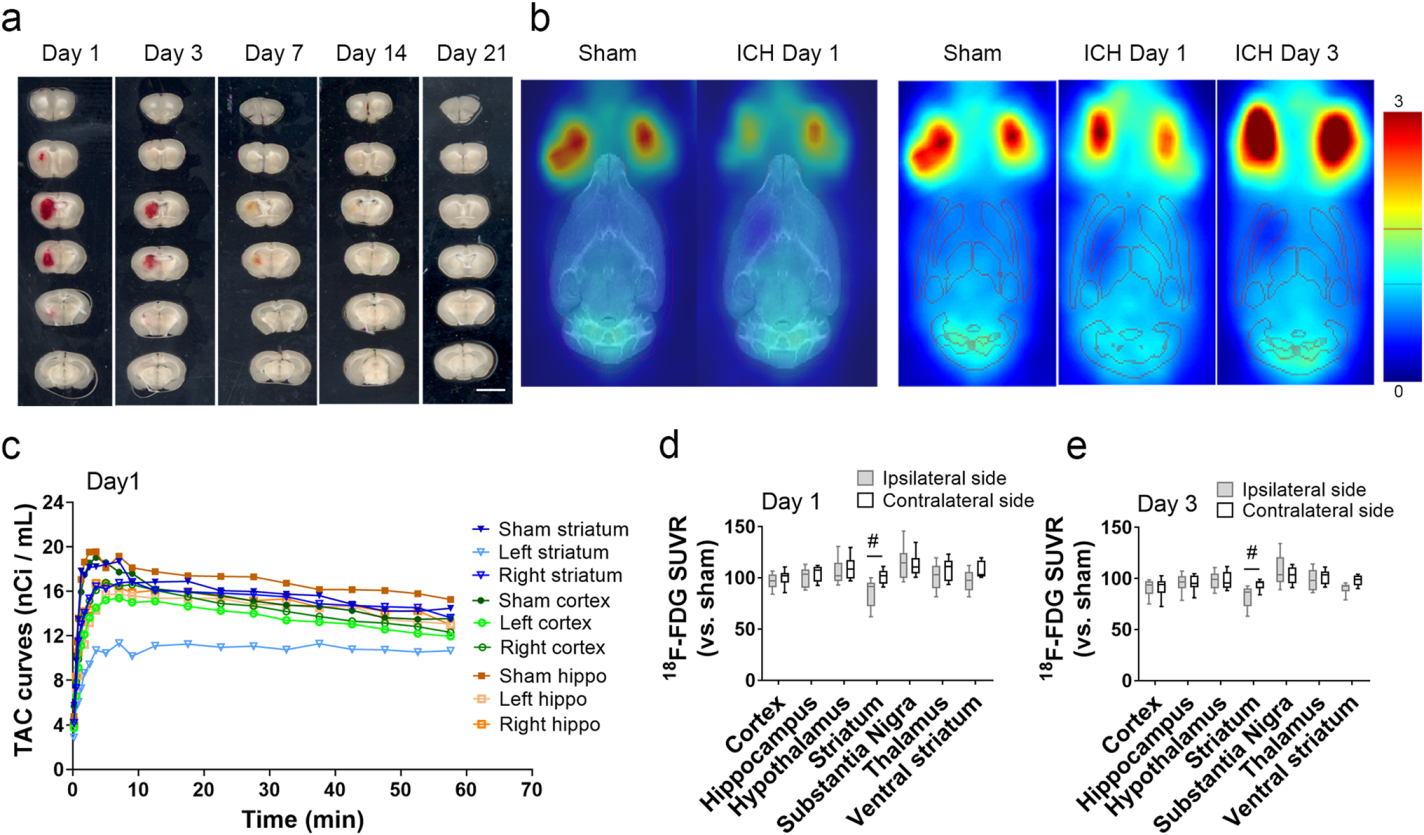
^18^F-FDG glucose uptake decreases in the ipsilateral striatum on days 1 and 3 after ICH. (**a**) Representative images of brain coronal slices show hematoma at different time points after collagenase-induced intracerebral hemorrhage (ICH). Brain slices were 1 mm thick. Scale bar: 500 µm. (**b**) Representative PET images of mice without (sham) or with ICH. Left panel: averaged PET images normalized to cerebellum (CB-ratio) for sham group and ICH group, fused with MRI reference template. Right panel: CB-ratio images fused with volume of interest (VOI) outlines to place brain regions. Red region shows nonspecific high activity area (blood in retro-orbital sinus). (**c**) Time activity curves (TAC) of striatum, cortex, and hippocampus (hippo) during 60 min of PET scanning on day 1 after ICH. n = 3. Each symbol represents the mean at that time point. (**d**, **e**) Average ^18^F-FDG standardized uptake value ratio (SUVR) at 40–60 min of PET scan on day 1 (**d**) and day 3 (**e**) after ICH. n = 9; #*p* < 0.05. Two-way ANOVA by hemisphere and brain region followed by Bonferroni post hoc test. Results are presented as box-and-whisker plots (the middle horizontal line within the box represents the median, boxes extend from the 25th to the 75th percentile, and the whiskers represent 95% confidence intervals).

Hematoma volume in the ipsilateral striatum decreased over time after ICH (Fig. 1a). At 1 day after collagenase administration, ^18^F-FDG uptake in the ipsilateral striatum was notably lower than that of sham-operated animals, as seen in both the averaged PET images and TAC curves (Fig. 1b, c). Evaluation of the TAC curves at 40–60 min of the PET scan showed that reduction of glucose metabolism in the ipsilateral striatum began on day 1 and persisted to day 3 post-ICH. The ^18^F-FDG SUVR as a percentage of that in the sham striatum was significantly lower in the ipsilateral than in the contralateral striatum on day 1 (85.03 ± 13.27% vs. 100.85 ± 7.19%, respectively, *p* < 0.05) and on day 3 (82.34 ± 10.52% vs. 93.62 ± 4.78%, respectively, *p* < 0.05; n = 9 per group). In contrast to the striatum, ICH did not cause a significant decrease in ^18^F-FDG uptake in other parts of the brain in the early stage after ICH (Fig. 1d, e).

### Glucose metabolism increases in the ipsilateral striatum in late stage of ICH

**Figure 2:**
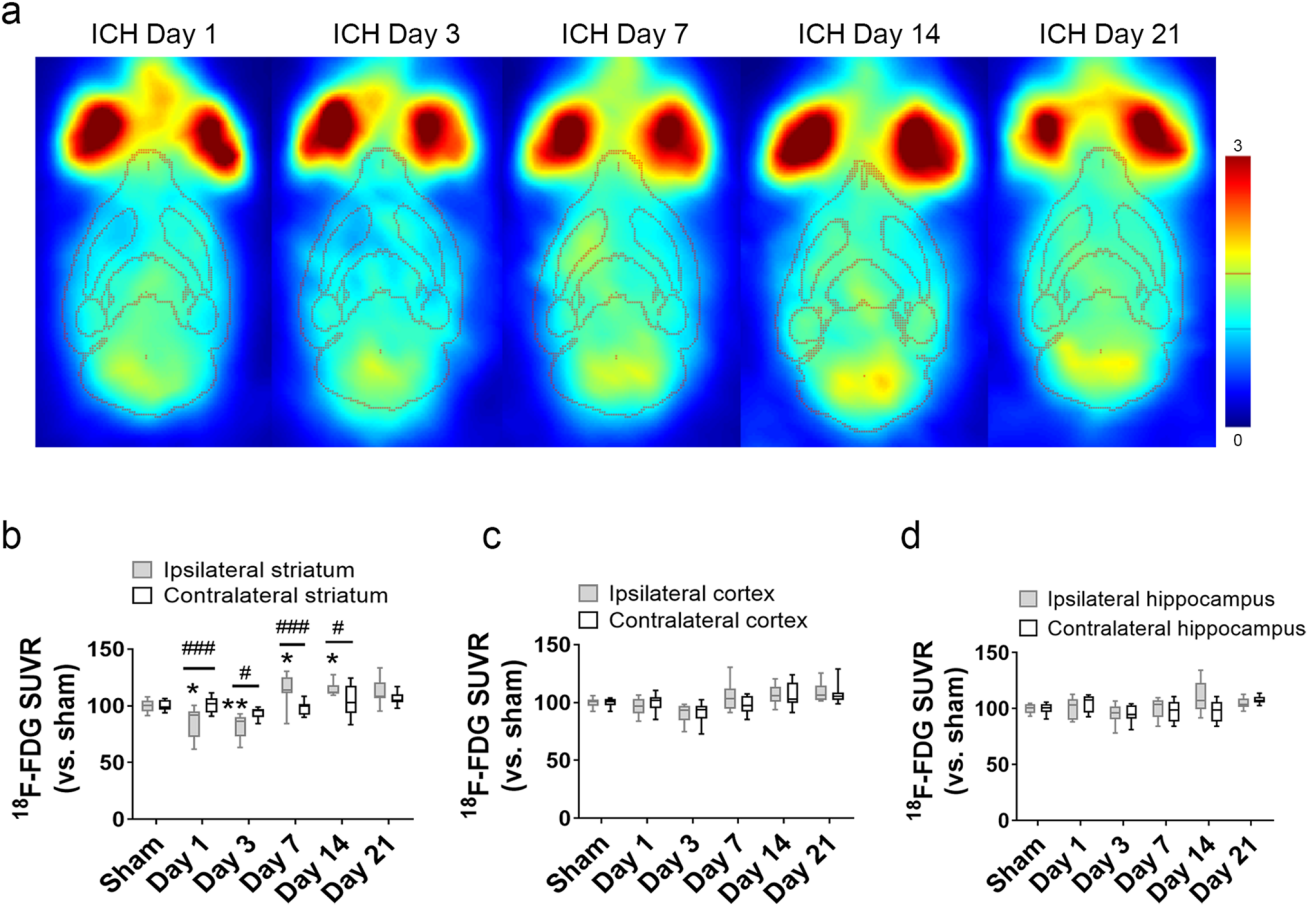
Glucose metabolism is increased in ipsilateral striatum on days 7 and 14 after ICH. (**a**) Representative PET images normalized to cerebellum at different time points after ICH, fused with volume of interest outlines to place brain regions. (**b**–**d**) Average ^18^F-FDG standardized uptake value ratio (SUVR) in striatum (**b**), cortex (**c**), and hippocampus (**d**) at different time points after ICH. n = 6 in sham group; n = 9 on days 1, 3, 7, and 14; n = 12 on day 21. *p < 0.05, **p < 0.01 versus sham group; #*p* < 0.05, ###*p* < 0.001 versus contralateral side. Two-way ANOVA with Bonferroni multiple comparison test. Data are presented as box and whisker plots (the middle horizontal line within the box represents the median, boxes extend from the 25th to the 75th percentile, and the whiskers represent 95% confidence intervals).

Although ^18^F-FDG uptake in the ipsilateral striatum was decreased on days 1 and 3 after ICH, it was elevated on days 7 and 14. The ^18^F-FDG SUVR as a percentage of that in the sham striatum was 114.98 ± 13.49% (ipsilateral) versus 98.25 ± 5.87% (contralateral; *p* < 0.001) on day 7 and 115.06 ± 5.71% (ipsilateral) versus 104.51 ± 13.50% (contralateral; *p* < 0.05) on day 14 (n = 9–12 per group; Fig. 2a, b). Compared to the contralateral hemisphere, neither ipsilateral cortex nor ipsilateral hippocampus differed in ^18^F-FDG uptake at any time point (Fig. 2c, d).

### Expression of GLUT1 decreases in the ipsilateral striatum after ICH

**Figure 3:**
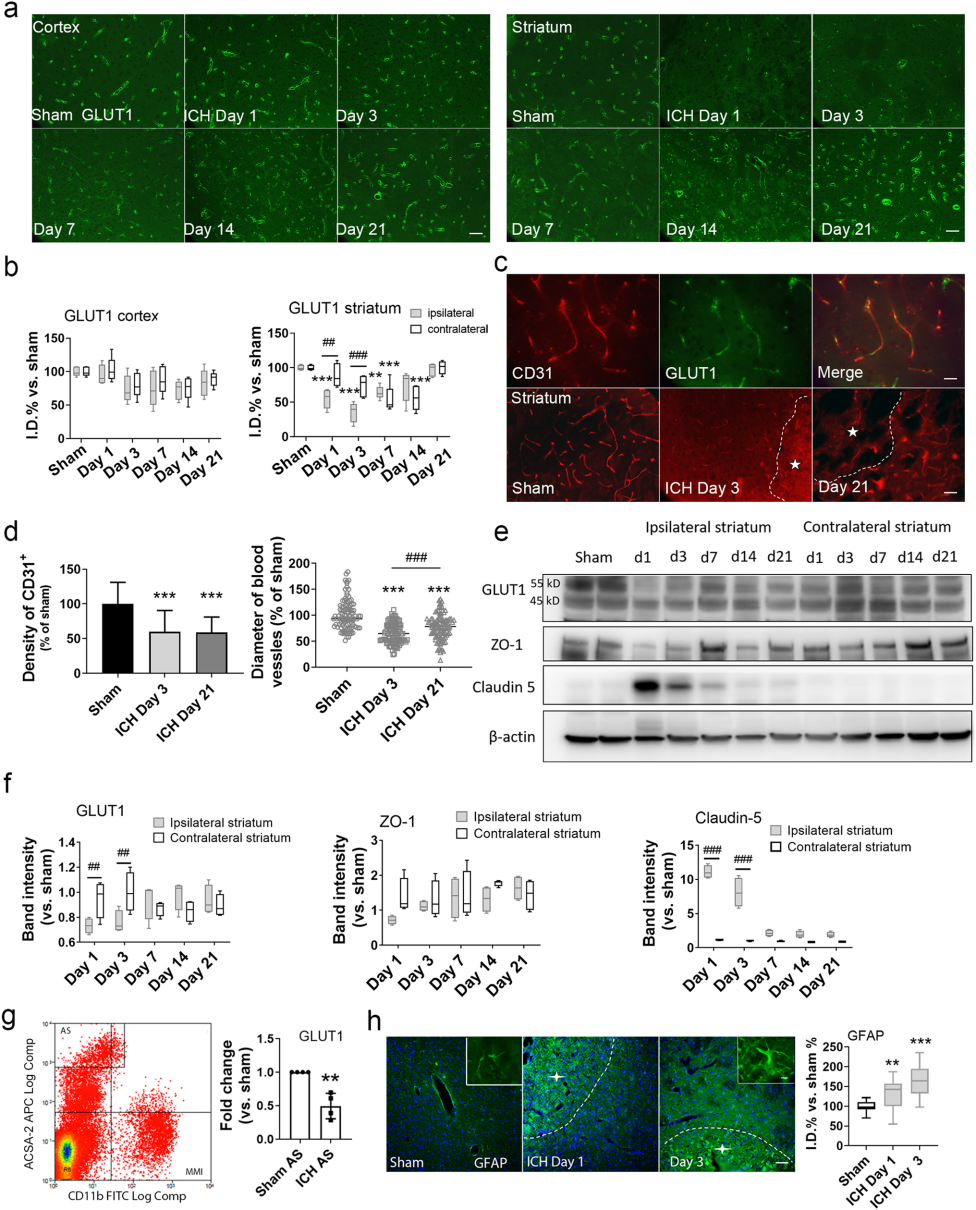
GLUT1 expression decreases in the ipsilateral striatum after ICH. (**a**, **b**) Representative images and quantification of GLUT1 (green) expression in the ipsilateral cortex (left panel) and ipsilateral striatum (right panel) after ICH. Scale bar: 50 µm. n = 5/group; **p < 0.01, ***p < 0.001 versus sham group; ###*p* < 0.001 versus contralateral side. Two-way ANOVA with Bonferroni multiple comparison test. (**c**) Representative images of CD31 (red) and GLUT1 (green) expression in the perihematomal region after ICH. The upper panels show the blood vessels under higher magnification. Scale bar, 25 µm. The lower panels show CD31 expression at days 3 and 21 after ICH. Scale bar, 50 µm. Stars in the outlined areas indicate the hemorrhagic core. (**d**) The density and diameter of blood vessels in ipsilateral striatum after ICH. n = 5/group; ***p < 0.001 versus sham group; ###*p* < 0.001 versus indicated group. One-way ANOVA with Bonferroni post hoc test. Data are presented as means ± SD. (**e**) Expression of GLUT1, ZO-1, and claudin-5 was evaluated by Western blotting at the indicated time points after ICH. The four images were from different gels. The samples derived from the same experiments and the gels were processed in parallel. To ensure the sensitivity of western blotting, we avoid to strip the membrane repeatedly. The membranes were cut before they were hybridized with the primary antibodies according to the ranges of their molecular weights. Raw images of the blots are presented in Supplementary Fig. 3. (**f**) Quantification of GLUT1, ZO-1, and degraded claudin-5 in striatum. n = 4/group; ##*p* < 0.01, ###*p* < 0.001; two-way ANOVA with Bonferroni multiple comparison test. Data are presented as box and whisker plots (the middle horizontal line within the box represents the median, boxes extend from the 25th to the 75th percentile, and the whiskers represent 95% confidence intervals). (**g**) Representative gating of CD11b-FITC^-^ / ACSA-2-APC^+^ astrocytes and fold change of GLUT1 mRNA in astrocytes at day 1 after ICH. n = 4, **p < 0.01 versus sham. AS, astrocytes; MMI, macrophage/microglia. (**h**) Representative images and quantification of GFAP (green) expression in the ipsilateral striatum on days 1 and 3 after ICH. Scale bar: 50 µm; inset: 10 µm. Stars in the outlined areas indicate the hemorrhagic core. n = 5/group; **p < 0.01, *** *p* < 0.001; one-way ANOVA with Bonferroni multiple comparison test. Data are presented as box and whisker plots (the middle horizontal line within the box represents the median, boxes extend from the 25th to the 75th percentile, and the whiskers represent 95% confidence intervals).

We used immunohistochemistry and Western blotting of brain tissue to evaluate the level of GLUT1, the brain’s primary glucose transporter. As expected, GLUT1 was expressed on the endothelium (Fig. 3a). Compared to levels in the sham group, GLUT1 was decreased in the ipsilateral striatum from day 1 to day 14 after ICH, but returned to normal level at day 21. The contralateral striatum also expressed a decreased GLUT1 level at days 7 and day 14. When compared with the contralateral striatum, the ipsilateral striatum showed significantly reduced GLUT1 expression on days 1 and 3 after ICH (n = 5, *p* < 0.01 and *p* < 0.001, respectively). In contrast to the ipsilateral striatum, the ipsilateral cortex did not show any change in GLUT1 at any time point after ICH (Fig. 3b).

Given that GLUT1 is expressed on endothelial cells and that blood vessels are damaged after ICH, we investigated whether the changes in GLUT1 are associated with loss of blood vessel integrity after ICH. We found that the density and diameter of CD31-positive blood vessels in the perihematomal region were decreased from day 3 until at least day 21 after ICH (Fig. 3c, d). We further evaluated the expression of GLUT1 and the tight junction proteins ZO-1 and claudin-5 by immunoblotting (Fig. 3e). GLUT1 expression was decreased on days 1 and 3 after ICH, consistent with our histological results. Expression of degraded claudin-5 was upregulated as early as day 1 after ICH. The expression of original claudin-5 was decreased on day 3 after ICH (Supplementary Fig. S1). We found no significant differences in ZO-1 level between the contralateral and the ipsilateral sides at any time point after ICH, although we noted a slight but nonsignificant decrease on day 1 after ICH (n = 4; Fig. 3f). These data indicate that expression of GLUT1 protein in brain is reduced after ICH and may be associated with loss of blood vessel integrity.

The 45 kD GLUT1 is expressed in the endfeet of astrocytes. We did not find a notable loss of 45 kD GLUT1 by western blot analysis after ICH, although the mRNA level of GLUT1 in astrocytes from peri-hematomal region decreased on day 1 after ICH (Fig. 3g). Reactive astrocytes with overexpression of GFAP started to accumulate around the hematomal area on day 1 after ICH (Fig. 3h).

### GLUT3 and GLUT5 increase in the ipsilateral striatum after ICH

**Figure 4:**
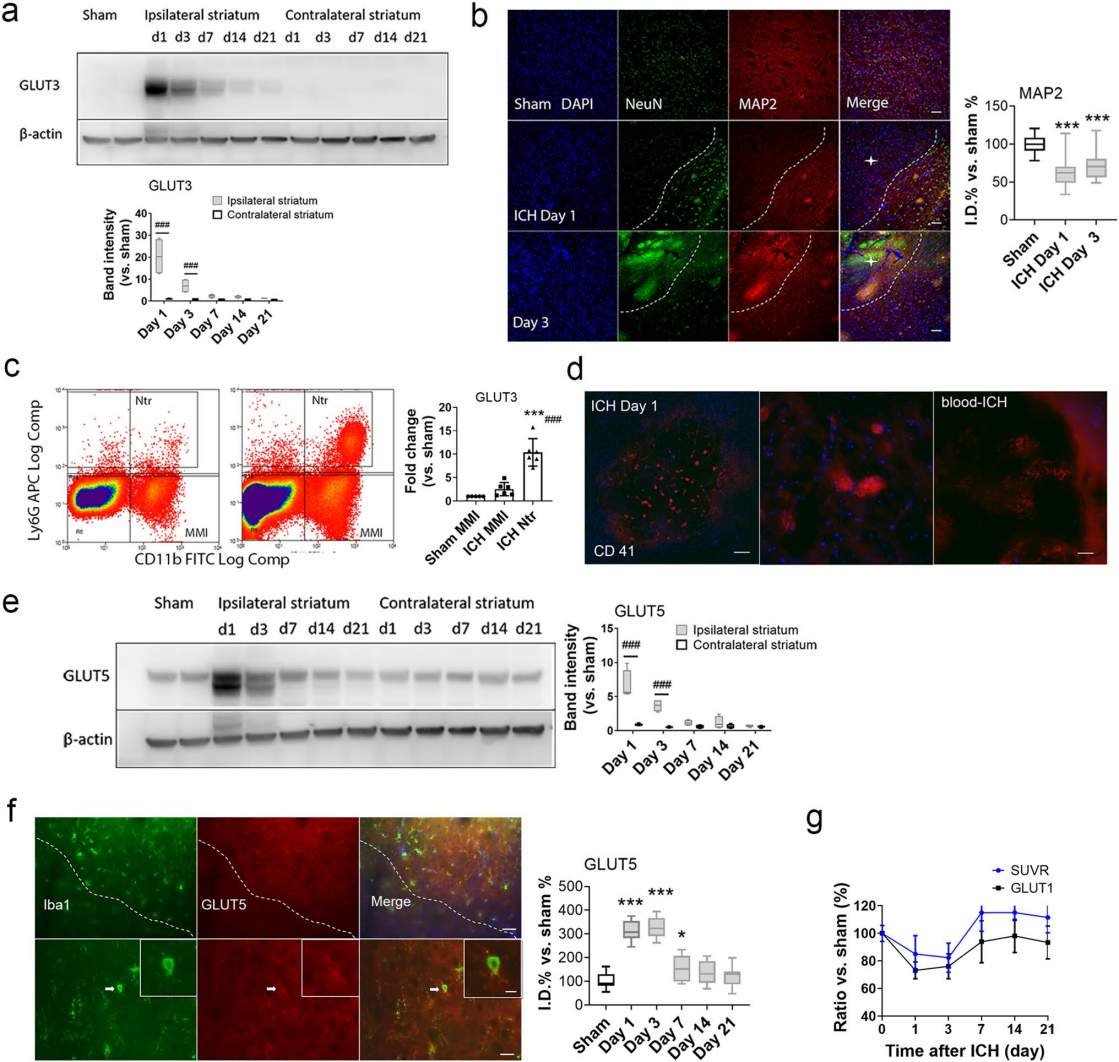
Expression of GLUT3 and GLUT5 increases in the ipsilateral striatum after ICH. (**a**) Representative Western blot (left) and quantification of GLUT3 expression (right) in striatum after ICH. n = 4/group. ###*p* < 0.001. Two-way ANOVA with Bonferroni post hoc test. The membrane was cut before it was hybridized with GLUT3 antibody according to molecular weight of GLUT3. Raw images of the blots are presented in Supplementary Fig. 3. (**b**) Representative images and quantification of MAP2 expression in the ipsilateral striatum on days 1 and 3 after ICH. Scale bar: 50 µm. Stars in the outlined areas indicate the hemorrhagic core. n = 5/group; *** *p* < 0.001; one-way ANOVA with Bonferroni multiple comparison test. (**c**) Representative gating of CD11b-FITC^+^ / Ly6G-APC^+^ neutrophils and CD11b-FITC^+^ / Ly6G-APC^-^ MMI and fold change of GLUT3 mRNA in cells at day 1 after ICH. n = 4, ***p < 0.001 versus sham MMI. *###p* < 0.001 versus ICH MMI. Ntr, neutrophils; MMI, macrophage/microglia. (**d**) Representative images of CD41 expression in the ipsilateral striatum on day 1 after collagenase-induced ICH and autologous blood-induced ICH. Red: CD41. Blue: DAPI. Scale bar: left 100 µm; right 25 µm. (**e**) Representative Western blot (left) and quantification of GLUT5 expression (right) in striatum after ICH. n = 4/group. ###*p* < 0.001. Two-way ANOVA with Bonferroni post hoc test The membrane was cut before it was hybridized with GLUT5 antibody according to molecular weight of GLUT5. Raw images of the blots are presented in Supplementary Fig. 3. (**f**) Brain sections were immunostained for Iba1 (green) and GLUT5 (red) to characterize microglia in the perihematomal region on day 3 after ICH. The area outlined in the lower left corner indicates the hematoma core. The arrow in the lower panel indicates one microglia under higher magnification. Insets show the same microglia under higher magnification. Scale bar: upper panel, 50 µm; lower panel, 25 µm; inset, 10 µm. Graph shows the quantification of GLUT5 expression in the ipsilateral striatum at multiple time points after ICH. n = 5/group. *p < 0.05, ***p < 0.001 versus sham group. (**g**) Comparison of standardized uptake value ratio on PET scan and relative GLUT1 level evaluated by Western blotting in the ipsilateral striatum at the indicated times after ICH. Pearson correlation test. Correlation coefficient *r* = 0.845, *p* = 0.034. Data are presented as mean ± SD at each time point.

To determine whether other glucose receptors affect the ^18^F-FDG glucose uptake on PET scans, we evaluated GLUT3 and GLUT5 expression at each time point after ICH. GLUT3 expression was significantly higher in the ipsilateral striatum than in the contralateral striatum on days 1 and 3 after ICH, and then gradually returned to baseline (Fig. 4a). We were unable to detect GLUT3 immunostaining on neurons successfully. We observed only diffuse GLUT3 signaling through the whole brain section (data not shown). However, histology did reveal that loss of neurons began on day 1 after ICH (Fig. 4b). To address whether infiltrated circulating cells such as neutrophils might influence the level of GLUT3, we collected neutrophils in the ipsilateral striatum by flow cytometry on day 1 after ICH. The GLUT1 mRNA level in the sorted neutrophils increased significantly more than that of the macrophage/microglia population (Fig. 4c). We also found that platelets, presumably as part of the repair process after ICH, accumulated in the hematomal lesion (Fig. 4d). GLUT5 was similarly upregulated in the ipsilateral striatum on days 1 and 3 (Fig. 4e). Histological results revealed that GLUT5 was located in microglia of the perihematomal region after ICH (Fig. 4f). GLUT5 expression in microglia was prominent on day 1 after ICH and gradually disappeared on day 14 after ICH (Supplementary Fig. S2).

Finally, we evaluated the correlation between SUVR and GLUT expression at different time points after ICH. We found that GLUT1 level significantly correlated with SUVR in the ipsilateral striatum after ICH (*r* = 0.845, *p* = 0.034; Fig. 4g). There was no correlation between GLUT3 or GLUT5 and SUVR (data not shown).

### ICH upregulates Iba1- and MPO-positive cells in the ipsilateral striatum

**Figure 5:**
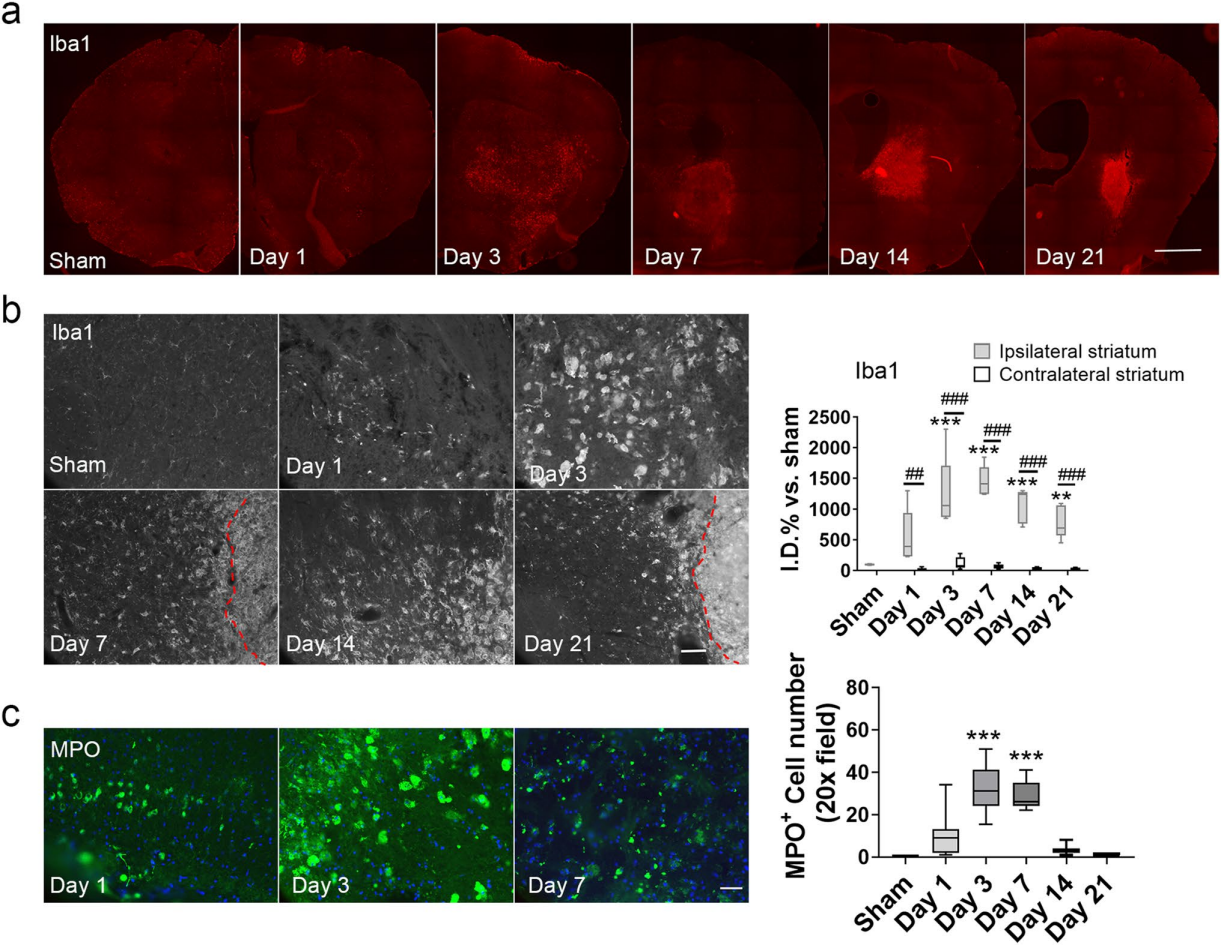
Iba1- and myeloperoxidase (MPO)-positive cells are heightened in ipsilateral striatum after ICH. (**a**) Representative images show ipsilateral brain sections stained for Iba1 (red) under low magnification at each time point after ICH. Scale bar: 500 µm. (**b**) Representative images (left) and quantification (right) of Iba1 (gray) expression in striatum at different time points after ICH. The outlined area indicates the hematoma core. Scale bar: 50 µm. n = 5/group. **p < 0.01, ***p < 0.001 versus sham group; ##*p* < 0.01, ###*p* < 0.001 versus contralateral side. Two-way ANOVA with Bonferroni post hoc test. (**c**) Representative images (left) and quantification (right) of MPO-positive cells (green) in the perihematomal region after ICH. Scale bar: 50 µm, blue: DAPI. n = 5/group. ***p < 0.01 versus sham group. One-way ANOVA with Bonferroni post hoc test.

To address whether activated microglia and infiltrating neutrophils/monocytes contribute to the increased ^18^F-FDG uptake on PET scans after ICH, we evaluated the numbers of Iba1-positive and MPO-positive cells after ICH. In the ipsilateral striatum, Iba1-positive cells started to accumulate around the perihematomal region on day 1 post-ICH (Fig. 5a). Moreover, the Iba1 expression was higher than that of the contralateral striatum at each time point assessed after ICH (Fig. 5b). Immunohistochemical analysis showed that MPO-positive cells in the ipsilateral striatum increased 1 day after ICH, peaked at day 3, and then declined toward 0 at day 14. The MPO-positive cells that infiltrated the brain at 1 day post-ICH were smaller than those observed on day 3, which exhibited the typical morphology of macrophages (Fig. 5c).

## Discussion

In this study, ^18^F-FDG-PET scans detected early glucose hypometabolism and later glucose hypermetabolism in the ipsilateral striatum after ICH. We did not observe compensatory glucose uptake in the contralateral striatum at any time point after ICH. Our results showed that the glucose hypometabolism was associated with a decrease in GLUT1 expression as well as damage to blood vessels, rather than to the briefly increased levels of GLUT3 and GLUT5. The activated microglia and infiltrating neutrophils/macrophages might contribute to the glucose hypermetabolism in the ipsilateral striatum during the late phase after ICH.

^18^F-FDG-PET imaging detects glucose hypometabolism in brain diseases that are correlated with microstructural abnormalities. For example, Alzheimer’s patients with cerebral amyloid angiopathy-related microbleeds exhibit glucose hypometabolism in the temporal lobe, whereas those without microbleeds do not^32^. Similarly, cerebral glucose metabolism is significantly lower in very low-birth-weight infants with low-grade intraventricular hemorrhage than in those without hemorrhage^33^. Our observation that ^18^F-FDG uptake was significantly decreased in ipsilateral striatum on days 1 and 3 after ICH, implies that the reduction is a result of broken blood vessels, oxygen deprivation, hematoma expansion, and cell death in the acute post-ICH period^1^. The data support previous studies showing rapid disruption in glucose metabolism after brain insults.

However, prior studies also revealed asymmetry of cerebral glucose metabolism after cerebral hemorrhage. Patients with subcortical aphasia or post-ICH pain exhibit diffused hypermetabolism in the contralateral cerebrum or thalamus^20,21^. The hypermetabolism might result from a pathophysiologic imbalance after ICH. A higher contralateral metabolic rate of glucose was shown to be associated with a better outcome in patients who experienced ischemic stroke^34^. Physical exercise increased glucose metabolism in the contralateral motor cortex and striatum of rats with ischemic stroke, leading to enhanced neural activity^35^. Thus, a compensatory increase in glucose metabolism in the contralateral hemisphere might protect neural function after ICH. Nevertheless, we did not observe increased ^18^F-FDG uptake in the contralateral striatum or in other cerebral regions in our model. It is possible that different species have variable styles of energy interaction. Additionally, the anatomic resolution of micro-PET scans in small rodents may not recapitulate the results of PET scanning in human brain.

GLUT1, the primary glucose transporter that transfers glucose from blood to brain, is present in two isoforms in brain: the 55 kD endothelial GLUT1 and the 45 kD astrocytic GLUT1^6^. GLUT1 expression is associated with the density and diameter of cerebral microvessels, as well as with cerebral ischemia^36,37^. Therefore, damage to blood–brain barrier integrity may be the cause of the observed reduction in GLUT1 expression at the early stage after ICH. While it is tempting to speculate that the decreased expression of GLUT1 is responsible for the decrease in glucose consumption, some decrease in GLUT1 could be tolerated because cerebral venous blood glucose is high relative to the transporter and remaining intact endothelial cells could have sufficient reserve capacity to partially compensate for the decreased GLUT1 expression. Moreover, it should be noted that mitochondria isolated from the perihematoma region of patients with ICH had impaired respiration^38^ and that CMRO_2_ is reduced in this region^39^. Thus, an alternative explanation is that impaired oxidative phosphorylation in perihematomal tissue or neuronal cell death decreases the glucose consumption. We cannot exclude that immediate tissue hypoxia and augmented anaerobic glycolysis preceding neuronal cell death would transiently increase tissue glucose consumption before our first PET measurement at one day.

We found that GLUT1 expression was higher in the ipsilateral striatum than in the contralateral striatum on days 7 and 14 after ICH and then recovered to baseline by day 21. However, blood vessel diameter and density remained low in the peri-hematomal region on day 21. In previous studies, mice that engaged in physical exercise displayed enhanced local neural activity along with increased GLUT1 expression^40,41^. Thus, the asynchronous upregulation of GLUT1 and blood vessel recovery most likely reflects both reconstruction of blood vessels and locally increased glucose demand from neural activity in the late phase after ICH. We also noticed that compared to sham group, the contralateral striatum expressed a decreased GLUT1 level on days 7 and 14 after ICH. Whether the lower GLUT1 expression in the contralateral striatum results in absent compensation in ^18^F-FDG uptake still needs further study. In addition, although endothelial GLUT1 and astrocytic GLUT1 are indistinguishable by immunohistochemistry, our results showed that GLUT1 mRNA expression decreased in the perihematomal astrocytes on day 1 after ICH. This decrease might account for some of the loss of glucose utilization in the ipsilateral striatum in the acute period after hemorrhage. Notably, reactive astrocytes demarcate the hematoma lesion and can be discerned by overexpression of GFAP along with time after ICH. Therefore, we cannot exclude the possibility that proliferation of astrocytes might also accompany increased GLUT1 expression on the endfeet in the late stage of ICH.

GLUT3 is highly expressed in neurons and transports glucose from the extracellular space^42^. Our data revealed a significant increase in GLUT3 expression in the ipsilateral striatum early after ICH, suggesting a rapid neuronal glucose demand in response to oxygen and glucose disruption. However, we have shown previously that necrosis, autophagy, apoptosis, and ferroptosis are involved in neuronal death in the acute period after ICH^31,43^. Therefore, even though investigators have reported that higher neuronal GLUT3 benefits neuronal function and learning in Alzheimer’s disease^44–46^, it seems less likely in our study. Neuronal death in the ipsilateral striatum certainly contributed to the glucose hypometabolism detected in ^18^F-FDG-PET imaging. Besides the main location in neurons, GLUT3 has been reported to be expressed in circulating cells such as neutrophils and platelets^47,48^. Our data confirmed that neutrophils, as one of the earliest infiltrated inflammatory cell after ICH, exhibited upregulated mRNA level of GLUT3. We also found that platelets, as prominent part of coagulation response to hemorrhage, accumulated in the hematomal lesion in both collagenase and blood-induced ICH model. Platelets have apparent levels of both GLUT1 and GLUT3 that could be involved in the glucose metabolism after activation during clotting responses^49,50^. We noticed that compared to the ipsilateral striatum, GLUT3 expression was not significantly detected in the undamaged contralateral hemisphere or in sham group by western blotting. Therefore, the infiltrated circulating cells and activated platelets from blood are likely to mainly account for the initial rapid increased in GLUT3 expression in the acute period after ICH. We observed that the GLUT3 level declined to the baseline rather than upregulating in the late stage of ICH. Whether neurons rely more on another energy source, such as blood-derived lactate^51^, than on cerebral glucose after ICH needs further investigation.

GLUT5 is present predominantly on microglia, and its ligand is fructose^52^. Fructose is generated from glucose not only via glycolysis under physiologic conditions, but also through the polyol pathway during hyperglycemia^53^. Our data support previous findings that activation of microglia increases GLUT5 expression^54^. However, why GLUT5 increases after brain injury is not yet known^52^. The level of intracerebral fructose is related to changes in intracerebral glucose^53^. Additional investigation may uncover a change in fructose concentration in the ipsilateral striatum after ICH. A recent study demonstrated that microglia utilize glutamine as an alternative metabolic fuel in the absence of glucose to maintain their immune surveillance function^55^. Hence, whether the change in GLUT5 expression after ICH reflects an integrated energy supply in microglia also warrants future investigation.

In recent years, ^18^F-FDG-PET/CT has shown promise in the management of infectious and inflammatory diseases, including fever, tuberculosis, vasculitis, and atherosclerotic plaque inflammation^56^. Several human studies have shown that most ^18^F-FDG accumulates in macrophage-rich areas of carotid artery atherosclerotic plaques^57–59^. Schroeter et al.^60^ exploited the selective binding of ^11^C-PK11195 to peripheral benzodiazepine receptors and used ^11^C-PK11195-PET to show that activated microglia and macrophages accumulate in the peri-infarct zone after permanent focal ischemia in rats. Further, in double-tracer experiments, they revealed that these reactive cells contribute to a nearly 60% increase in the ^18^F-FDG metabolic rate. In a mouse ICH study, resident microglia and infiltrating neutrophils/monocytes elicited secondary inflammation and hematoma resolution. These cells accumulated in the ipsilateral striatum for at least 21 days after ICH. Therefore, our results also suggest that increased inflammatory cell activity contributes to the increased energy demand after ICH.

Finally, several limitations to this study need to be acknowledged. First, we did not investigate ^18^F-FDG uptake at very early times, such as in the first few hours after ICH. Song et al.^61^ previously reported that rat with subarachnoid hemorrhage (SAH) showed decreasing uptake in lesion area correlating with neuronal death and increased HO-1 expression. However, the whole brain ^18^F-FDG SUV was elevated at 3 h and 12 h after SAH. We cannot exclude the possibility that the contralateral hemisphere might compensate for glucose metabolism at very early time points. Second, we used collagenase rather than autologous blood injection to induce ICH in order to mimic blood vessel rupture. Both models of ICH have limitations^62^. PET imaging of cats with autologous blood-induced ICH showed hypometabolism from 2 to 12 h after ICH that steadily recovered at 24 and 48 h^19^. Variation among species and different animal ICH models might affect ^18^F-FDG-PET results. Third, we did not evaluate other sources of energy, such as lactate, pyruvate, and lipids that might affect ^18^F-FDG uptake and GLUT expression after ICH^63,64^. And lastly, the influence of hypoglycemia or hyperglycemia on cerebral glucose metabolism after ICH should be considered.

In summary, we used ^18^F-FDG-PET scans to assess glucose metabolism and determined GLUT expression at multiple time points in a mouse ICH model. This study reflected the cellular pathologic process of glucose metabolism after ICH and outlined possible clinical implications for glucose management.

## Supplementary Information

Supplementary Information.
