# Supplementary material for: Analysis of glucose metabolism by 18F-FDG-PET imaging and glucose transporter expression in a mouse model of intracerebral hemorrhage
Source: Sci Rep. 2021 May 25;11:10885. doi: 10.1038/s41598-021-90216-4 (PMC8149426; doi:10.1038/s41598-021-90216-4)
Supplement: Supplementary file 1 — Supplementary Information. [file 41598_2021_90216_MOESM1_ESM.docx]

**Supplementary information for**

**Title: Analysis of Glucose Metabolism by ^18^F-FDG-PET Imaging and Glucose Transporter Expression in a Mouse Model of Intracerebral Hemorrhage**

Xiaoning Han^1^, Honglei Ren^1^, Ayon Nandi^2^, Xuanjia Fan^1^, Raymond C. Koehler^1^

^1^Department of Anesthesiology and Critical Care Medicine, Johns Hopkins University, School of Medicine, Baltimore, MD 21205, United States

^2^Division of Nuclear Medicine and Molecular Imaging, The Russell H. Morgan Department of Radiology and Radiological Science, Johns Hopkins University, School of Medicine, Baltimore, MD 21205, United States

This file includes four supplementary figures.


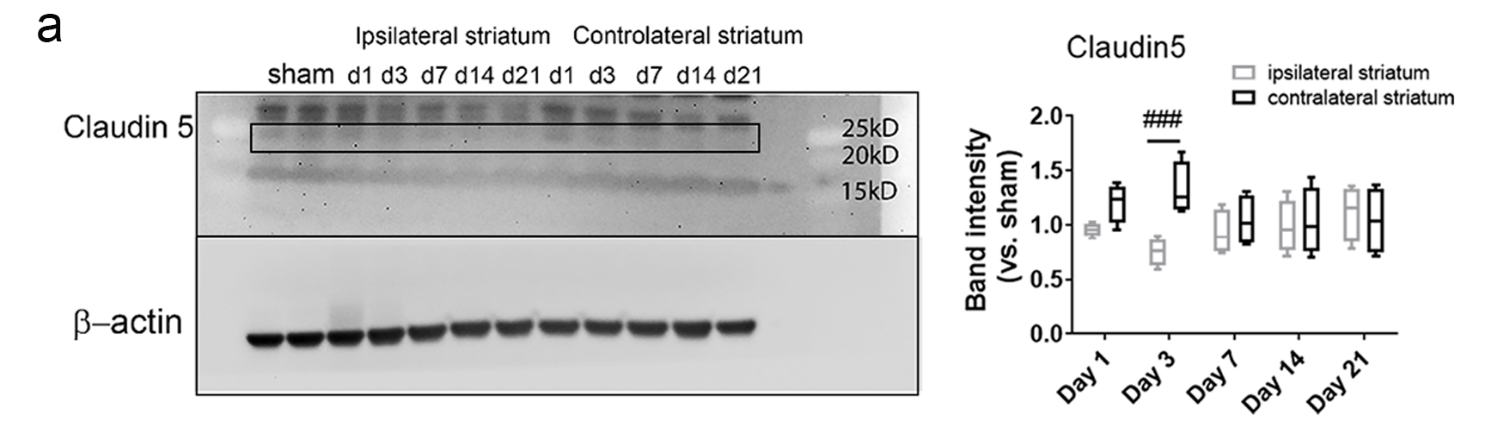


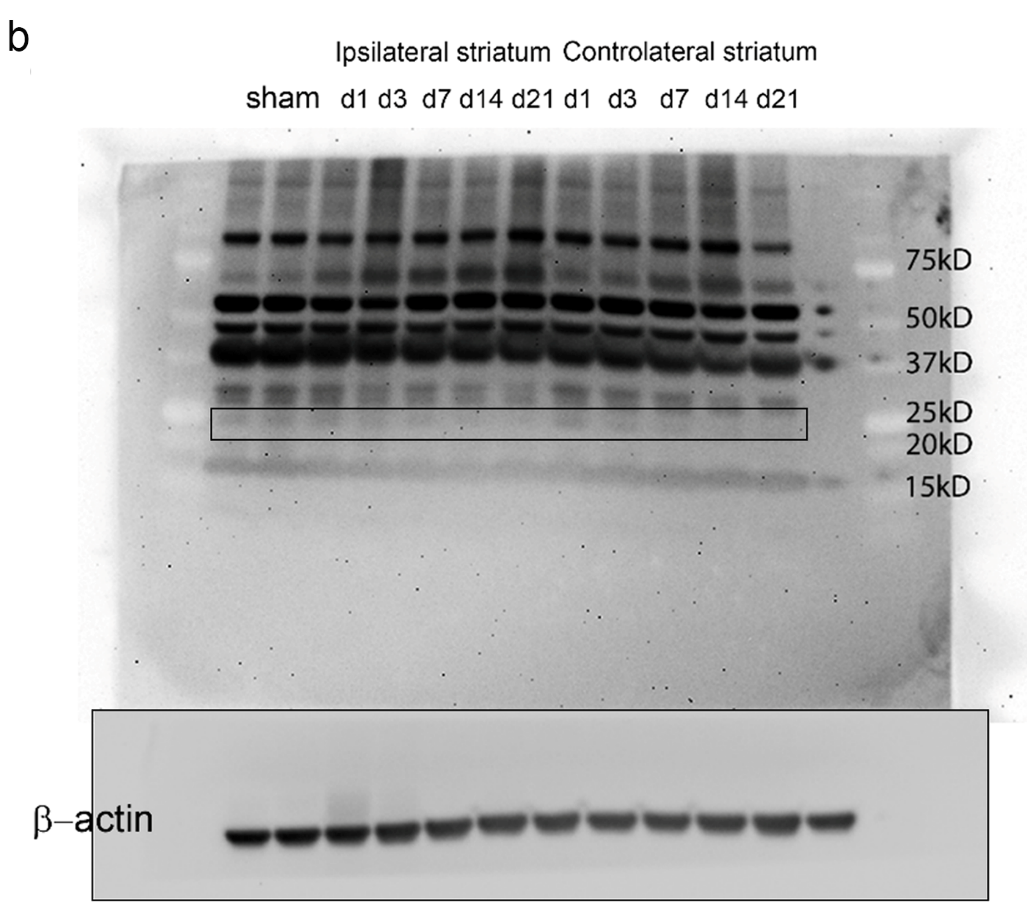


**Supplementary Figure 1.** Representative western blot and quantification of Claudin 5 expression in striatum after ICH. (a) Representative cropped gel of rabbit anti- claudin 5 antibody that detected the initial band of claudin 5 between 20 kD and 25 kD (outlined). n =4/group. ###*p*<0.001. Two-way ANOVA with Bonferroni post hoc test. Data are presented as box and whisker plots (the middle horizontal line within the box represents the median, boxes extend from the 25th to the 75th percentile, and the whiskers represent 95% confidence intervals). (b) Full length membrane for (a) showed the rabbit anti claudin 5.

**
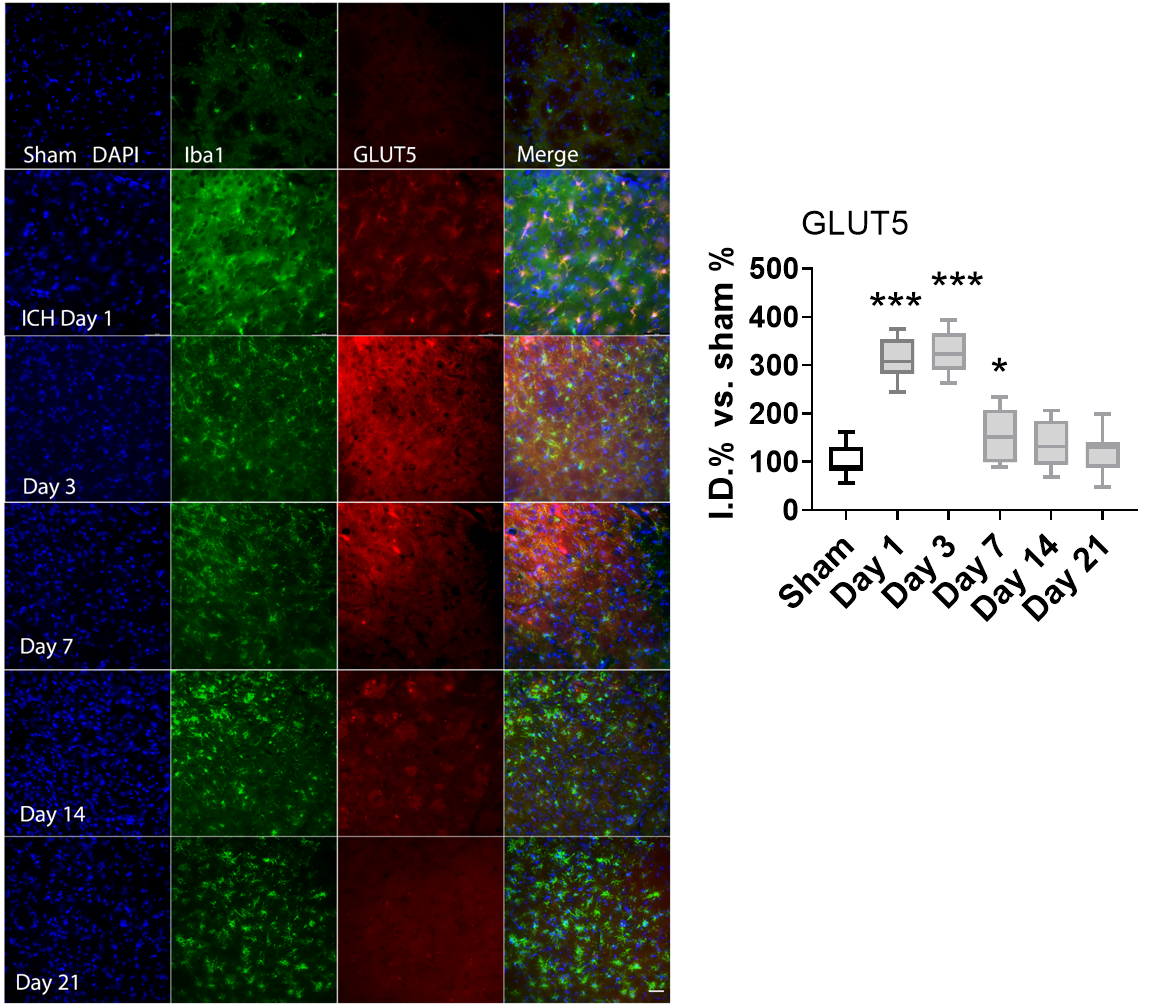
Supplementary Figure 2.** Representative images and quantification of GLUT5 in the ipsilateral striatum after ICH. DAPI, blue; Iba1, green; GLUT5, red; Scale bar: 25 µm. n = 5/group; **p*<0.05, ****p*<0.001 versus sham group; one-way ANOVA with Bonferroni multiple comparison test. Data are presented as box and whisker plots (the middle horizontal line within the box represents the median, boxes extend from the 25th to the 75th percentile, and the whiskers represent 95% confidence intervals).


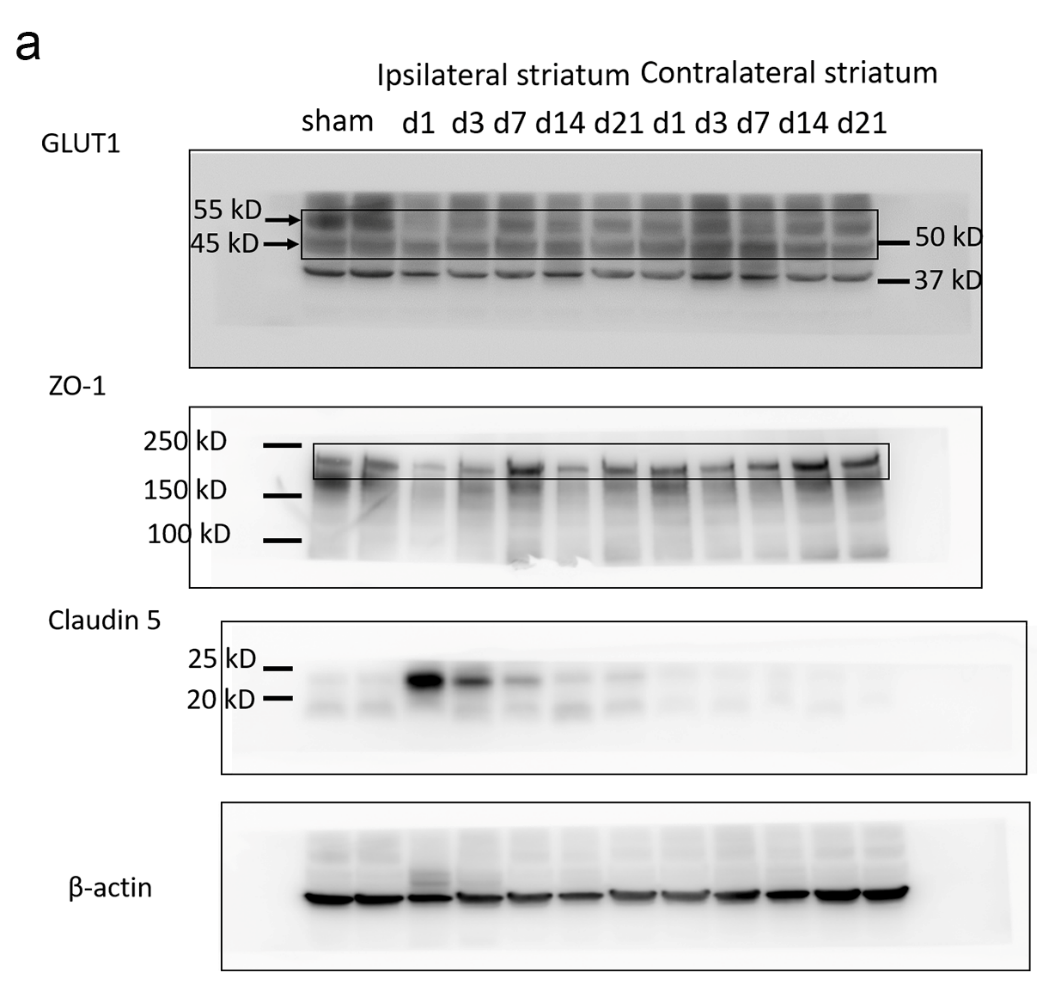

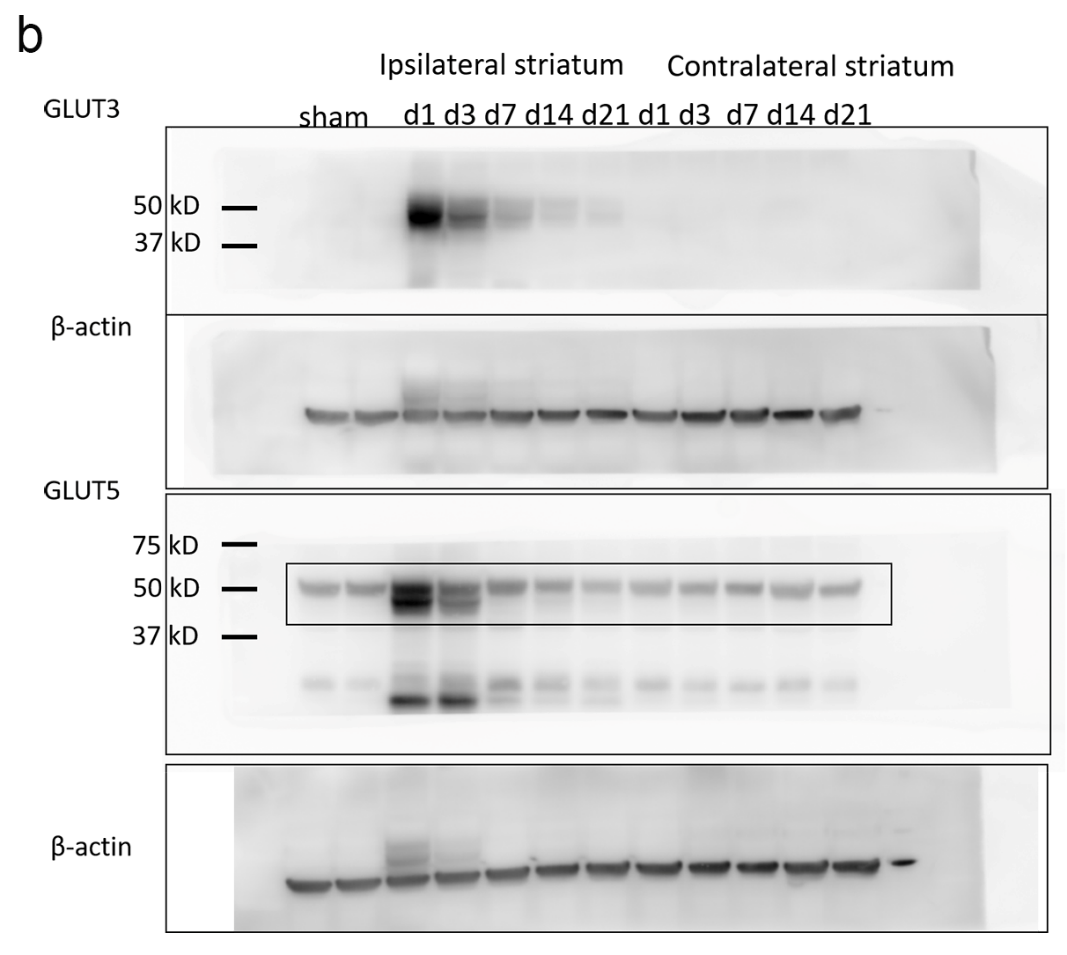


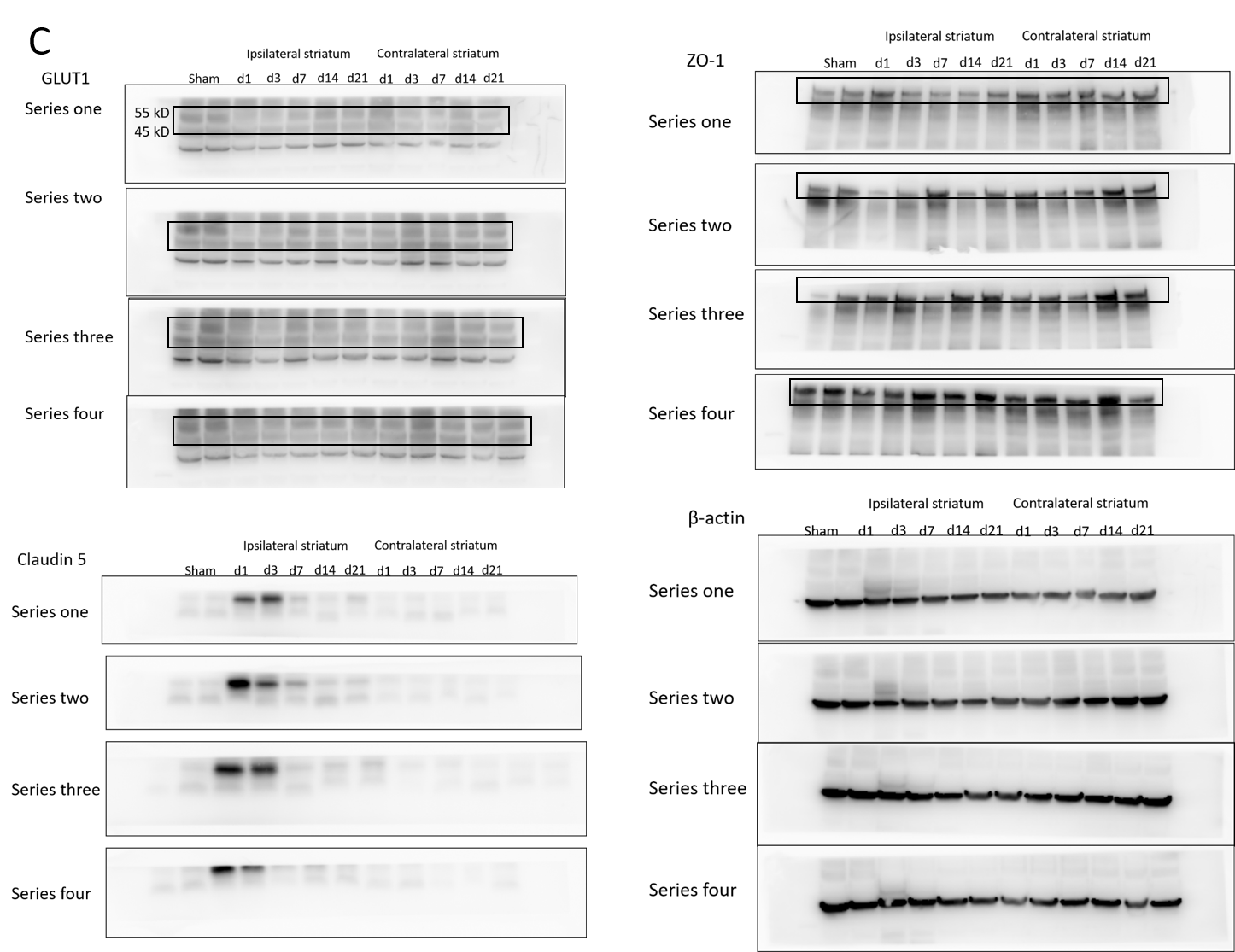


**
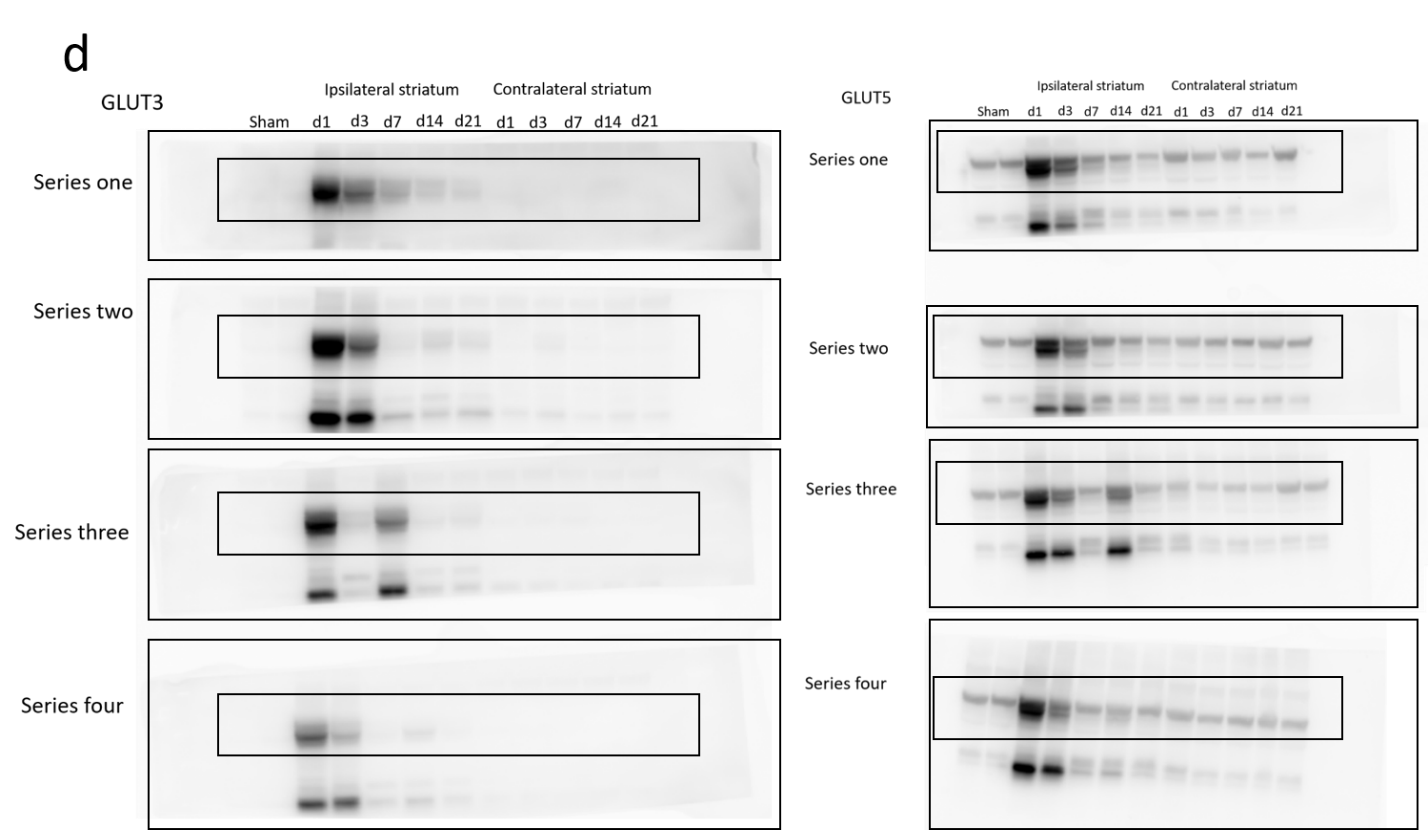
**

**
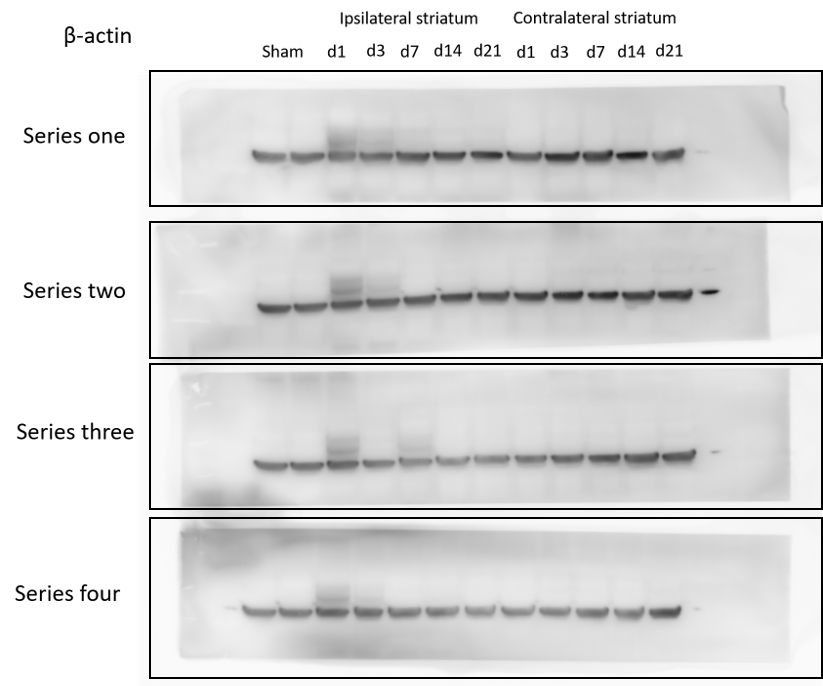
**

**Supplementary Figure 3.** Original western blot gels for Figures in the main article. Target blots were outlined. (a) Original western blot gels for the Figure 3e. The four images in Figure 3e were from different gels. The samples derived from the same experiments and the gels were processed in parallel. To ensure the sensitivity of western blotting, we avoid to strip the membrane repeatedly. The membranes were cut before they were hybridized with the primary antibodies according to the ranges of their molecular weight. Our previous publication have detected the molecular weight of ZO-1 antibody and Claudin 5 antibody.^29^ (b) Original western blot gels for the Figure 4a (upper GLUT3 and β-actin) and the Figure 4d (lower GLUT5 and β-actin). Figure 4a and Figure 4d were from different gels. The samples for Figure 4a and Figure 4d derived from the same experiments and the gels were processed in parallel. The membrane was cut before it was hybridized with the primary antibody according to the ranges of its molecular weight. (c) Raw images of all western blots for Figure 3e. (d) Raw images of all western blots for Figure 4a and 4d.


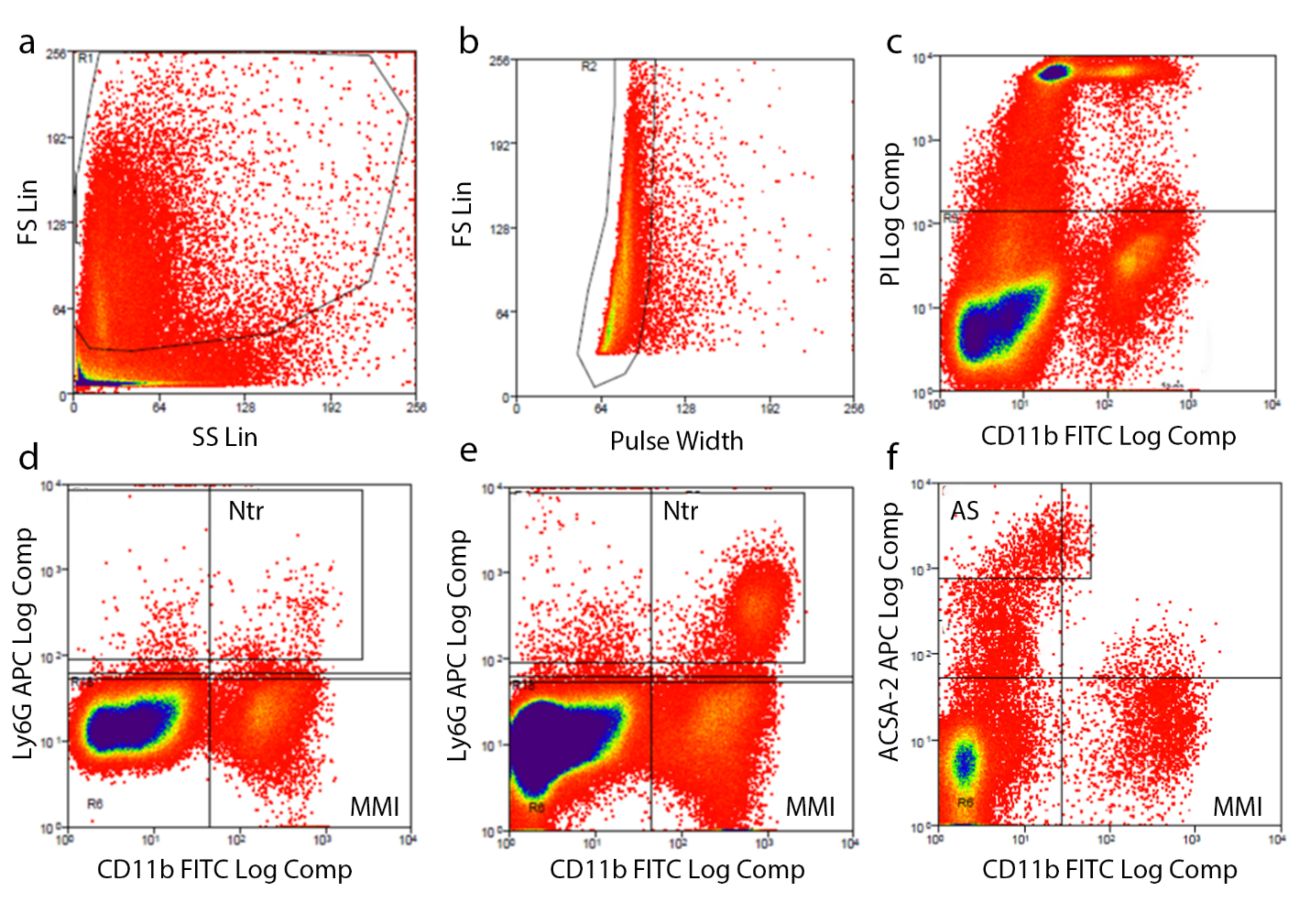


**Supplementary Figure 4**. Gating cells by flow cytometry on day 1 after ICH. (a) Representative gating of all cells. (b) Gating of singlets. (c) Gating of live cells. (d) Gating of CD11b-FITC^+^ / Ly6G-APC^+^ neutrophils and CD11b-FITC^+^ / Ly6G-APC^-^ Macrophages/microglia in sham group. (e) Gating of CD11b-FITC^+^ / Ly6G-APC^+^ neutrophils and CD11b-FITC^+^ / Ly6G-APC^-^ Macrophages/microglia in ICH group. (f) Gating of CD11b-FITC^-^ / ACSA-2-APC^+^ astrocytes and CD11b-FITC^+^ / ACSA-2-APC^-^ Macrophages/microglia in ICH group.
